# 103 Implementation of a physical activity referral scheme in the German healthcare system: A qualitative analysis of implementation success

**DOI:** 10.1093/eurpub/ckae114.079

**Published:** 2024-09-26

**Authors:** Sarah Klamroth, Eva Lorenz, Inga Naber, Eriselda Mino, Anja Weissenfels, Peter Gelius, Karim Abu-Omar, Wolfgang Geidl, Klaus Pfeifer

**Affiliations:** Friedrich-Alexander-Universität Erlangen-Nürnberg, Germany; Friedrich-Alexander-Universität Erlangen-Nürnberg, Germany; Friedrich-Alexander-Universität Erlangen-Nürnberg, Germany; Friedrich-Alexander-Universität Erlangen-Nürnberg, Germany; Friedrich-Alexander-Universität Erlangen-Nürnberg, Germany; Université de Lausanne, Switzerland; Friedrich-Alexander-Universität Erlangen-Nürnberg, Germany; Friedrich-Alexander-Universität Erlangen-Nürnberg, Germany

## Abstract

**Purpose:**

The BewegtVersorgt project is the first study to implement a physical activity referral scheme (PARS) in the German healthcare system. The PARS aims to increase physical activity (PA) levels among persons with non-communicable diseases and includes a brief PA advice by physicians and an individualized PA promotion by physical therapists or sports therapists (PARS group). In a pragmatic cluster-randomized controlled trial, the PARS group is compared to a group receiving PA advice only (PAA). Based on the RE-AIM framework, this qualitative study evaluates specific dimensions of implementation success of the PARS, all from health professionals’ perspective.

**Methods:**

We conducted semi-structured interviews (n = 13) with physicians and medical assistants from eight (out of 17) medical practices and with five therapists (out of 14 therapy practices). Following the principles of intensity case sampling, the practices were purposefully selected based on the number of participants treated and dropped-out. The interview transcripts were analyzed using thematic analysis, combining deductive and inductive category building.

**Results:**

Most health professionals report that they would already address PA promotion in some way and see this as part of their job, but in different roles. The majority of interviewees believes that both intervention types (PARS, PAA) have added value for the participants but the individual approach in the PARS group was seen as more advantageous by physicians (adoption). While most of the physicians did not use techniques of motivational interviewing (MI) for PA advice, therapists largely delivered the individualized PA promotion as intended and integrated MI. Both, therapists and physicians faced barriers with integrating the process into everyday practice and during intervention delivery (implementation). Most interviewees advocate a sustainable implementation of the program, taking into account adjustments to the intervention concept as well as structural and system-related changes (maintenance).

**Conclusions:**

Given the intervention adjustments and barriers faced with the implementation in routine care, implementation success was only partially achieved. These results, in combination with the evaluation of all dimensions of implementation success, will help to adapt the PARS to be more suitable for integration into the German healthcare system.

**Funding Source:**

Funded by the German Federal Ministry of Health.

